# Supplementary material for: Scalable parallel and distributed simulation of an epidemic on a graph
Source: PLoS One. 2023 Sep 29;18(9):e0291871. doi: 10.1371/journal.pone.0291871 (PMC10540973; doi:10.1371/journal.pone.0291871)
Supplement: S1 File — (PDF) [file pone.0291871.s001.pdf]

Supporting information

S1 Appendix.

7 Implementation

We present in this section our implementation of the algorithm in the form of pseudocode.

7.1 Data Structures

We start by defining the data structures and the operations they support.

7.1.1 Border

During initialization, a lookup table, referred to as the *border*, is constructed on each process P and will be held fixed throughout the simulation instance. Consider a relational database with the schema in table 2.

Table 2. Border data structure on Process P.

other_process (OP)	local_vertex (LV)	num_neighbors (NN)
Q	v	$n_{Q \to v} = \lvert Q \cap \mathcal{N}(v) \rvert$

The data structure in table 2 allows us to find efficiently

- all local vertices on P that have neighbors on process Q;
- all processes that have neighbors of local vertex v;
- the number of neighbors of local vertex v on Q.

This data structure supports the following operations:

- select⟨*colnames*⟩(*values*): Returns all rows whose *colnames* attributes have values *values*.

- project⟨*colnames*⟩(): Returns *colnames* attributes of all rows, with duplicates removed.

We assume indexes are built on "other_process" and "local_vertex" columns, meaning that select⟨OP⟩(Q) and select⟨LV⟩(v) do not require a full scan through their corresponding columns. Select⟨OP⟩(Q) returns $\mathcal{O}(|V|)$ rows. Select⟨LV⟩(v) returns $\mathcal{O}(M)$ rows. Because (other_process, local_vertex) forms a composite primary key, any projection after selection by OP or LV requires no deduplication. Project⟨OP⟩ without any previous selection does require deduplication, which has complexity $\mathcal{O}(M)$, thanks to the index on column OP.

7.1.2 Queue

Each process P maintains a min-heap of (scheduled_time, event) pairs, sorted based on the scheduled time of occurrence. Suppose the queue has size S before the operation takes place. It supports the following operations:

- push(*scheduled_time, event*): Adds *event* to the queue with *scheduled_time*. $\mathcal{O}(\log S)$ complexity. No-op if *event* is already in queue.

- remove(*event*): Removes *event* from queue, while maintaining the heap property. $\mathcal{O}(\log S)$ complexity. No-op if *event* is not in queue.

- fix(*scheduled_time, event*): Updates the original scheduled time of *event* to *scheduled_time*, and restores the heap property afterwards. $\mathcal{O}(\log S)$ complexity. No-op if *event* is not in queue.

- peek(): Returns the (t, event) pair at the top of the queue without modifying the queue. $\mathcal{O}(1)$ complexity. Undefined behavior if the queue is empty.

- size(): Returns the number of pairs in the queue. $\mathcal{O}(1)$ complexity.

7.1.3 Outbox/Inbox

If a vertex v on process P has neighbors on process Q, we say v is bordering Q. We let $n_{P\to Q}$ be the number of infected vertices on P bordering Q, i.e.,

$$n_{P\to Q} = |\{\, v \in P \mid v.\text{state}{=}\text{INF}, \exists u \in Q, (u,v) \in E \,\}|. \tag{14}$$

The collection $\{\, n_{P\to Q} \mid Q \in \mathcal{N}(P) \,\}$ is the *outbox* of the process P. The collection $\{\, n_{Q\to P} \mid Q \in \mathcal{N}(P) \,\}$ is the *inbox* of process P. Upon the execution of infection or recovery events on P, the variables in the outbox will be incremented or decremented accordingly to maintain the invariant in eq. (14).

7.1.4 Channel

We assume that each process P has access to the following operations for communication:

- ***StartSend***(Q, *val*): Puts the value *val* in the (P, Q) channel and returns immediately.

- ***Recv***(Q): Retrieves and returns the value in channel (Q, P) if there is any. Otherwise, blocks until a value enters channel (Q, P), retrieves it, and returns it.

- ***FinishSend***(Q): Returns immediately if the channel (P, Q) is empty. Otherwise, blocks until the channel (P, Q) becomes empty.

The corresponding APIs in MPI [38] are listed below:

- `MPI_Isend(const void *buf, int count, MPI_Datatype datatype,`
  `int dest, int tag, MPI_Comm comm, MPI_Request *request)`
- `MPI_Recv(void *buf, int count, MPI_Datatype datatype,`
  `int source, int tag, MPI_Comm comm, MPI_Status *status)`
- `MPI_Wait(MPI_Request *request, MPI_Status *status)`

7.2 Procedures

We assume we have the following subroutines:

- GetProcess(v): Returns the process that vertex v is on. $\mathcal{O}(1)$ complexity.
- Exp(λ): Generates a random number following an exponential distribution with rate λ. $\mathcal{O}(1)$ complexity.

We first present the algorithmic components, which will be combined into the ParSim algorithm.

7.2.1 Upon the Recovery of Vertex v on Process P

We present the procedure call upon the recovery of vertex v on process P, shown in algorithm 1.

Algorithm 1 OnRecovery

procedure OnRecovery(t, v, P, queue, border)
  v.state $\leftarrow$ SUS
  queue.remove(Rec(v))
  for u in v.neighbors **do**
    if GetProcess(u) $= P$ **then**
      if u.state = INF **then**
        queue.push(t+Exp(β), Inf($u \to v$))
      else
        queue.remove(Inf($v \to u$))
      end if
    end if
  end for
  for (Q, n) in border.select⟨LV⟩(v).project⟨OP, NN⟩() **do**
    $n_{P\to Q} \leftarrow n_{P\to Q} - 1$
    if $n_{Q\to P} \neq 0$ **then**
      queue.push(t+Exp($n \frac{n_{Q\to P}}{N_{Q\to P}} \beta$), Inf($Q \to v$))
    end if
  end for
end procedure

7.2.2 Upon the Infection of Vertex v on Process P

We present the procedure call upon the infection of vertex v on process P in algorithm 2. It closely mirrors its counterpart for recovery.

Algorithm 2 OnInfection

```
procedure OnInfection(t, v, P, queue, border)
    v.state ← INF
    queue.push(t+Exp(γ), Rec(v))
    for u in v.neighbors do
        if GetProcess(u) = P then
            if u.state = INF then
                queue.remove(Inf(u → v))
            else
                queue.push(t+Exp(β), Inf(v → u))
            end if
        end if
    end for
    for Q in border.select⟨LV⟩(v).project⟨OP⟩() do
        n_{P→Q} ← n_{P→Q} + 1
        queue.remove(Inf(Q → v))
    end for
end procedure
```

7.2.3 Upon Receiving Updated Inbox

Upon receiving the updated inbox $\{\, n_{Q\to P} \mid Q \in \mathcal{N}(P) \,\}$, infection events of the form Inf($Q \to v$) generated using the old inbox values need to be renewed, as shown in algorithm 3. The parameter epoch is the time when the current epoch starts. $n^*_{Q\to P}$ is the updated inbox value.

7.2.4 ParSim

We assume that each process P has access to a serialized version of its assigned section of the adjacency list, with which the graph and the initial vertex state assignment can be loaded into memory using load_graph(). The procedures for initializing the queue (init_queue) and the border (init_border) are straightforward and thus omitted. The subroutine for initialization is shown in algorithm 4.

The algorithm on each process P proceeds in rounds. In each round, the time in the simulated world (stored in variable *clock*) gets incremented by Δ until the desired horizon H is met, after which the algorithm terminates. The main ParSim algorithm is shown in algorithm 5.

S2 Appendix.

8 Load Balancing

In this section, we study different ways of graph partitioning and explore their impact on load balancing. We want to gain evidence that load balancing causes the different behaviors between fig. 6 and fig. 10.

In addition to uniform partitioning of er100k and METIS partitioning of lj4m in the main text, we conduct METIS partitioning of er100k and uniform partitioning of lj4m. Their timing information is shown in figs. 14 and 15.

We collect and plot the coefficients of variation of process computation times. We collect the time spent computing U_i^r on process P_i in the r-th repeated experiment for

Algorithm 3 OnRecv

procedure OnRecv(P, $n^*_{Q\to P}$, epoch, queue, border)
    if $n^*_{Q\to P} = n_{Q\to P}$ **then**
        return
    end if
    if $n^*_{Q\to P} = 0$ **then**
        for v in border.select⟨OP⟩(Q).project⟨LV⟩() **do**
            if v.state = SUS **then**
                queue.remove(Inf($Q \to v$))
            end if
        end for
    else
        for (v, n) in border.select⟨OP⟩(Q).project⟨LV, NN⟩() **do**
            if v.state = SUS **then**
                if $n_{Q\to P} = 0$ **then**
                    queue.push(epoch + Exp($n\frac{n^*_{Q\to P}}{N_{Q\to P}}\beta$), Inf($Q \to v$))
                else
                    queue.fix(epoch + Exp($n\frac{n^*_{Q\to P}}{N_{Q\to P}}\beta$), Inf($Q \to v$))
                end if
            end if
        end for
    end if
    $n_{Q\to P} \leftarrow n^*_{Q\to P}$
end procedure

Algorithm 4 ParSim: Init

procedure Init
    load_graph()
    init_border()
    init_queue()
    for Q in border.project⟨OP⟩() **do**
        $n_{Q\to P} \leftarrow 0, n_{P\to Q} \leftarrow 0$
        for v in border.select⟨OP⟩(Q).project⟨LV⟩() **do**
            if v.state = INF **then**
                $n_{P\to Q} \leftarrow n_{P\to Q} + 1$
            end if
        end for
    end for
end procedure

Algorithm 5 ParSim: Run

```
procedure Run(P, queue, border, Δ, H)
    clock ← 0
    while clock ≤ H do
        for Q in border.project⟨OP⟩() do
            StartSend(Q, n_{P→Q})
        end for
        for Q in border.project⟨OP⟩() do
            n*_{Q→P} ← Recv(Q)
            OnRecv(P, n*_{Q→P}, clock, queue, border)
        end for
        while queue.size() do
            t, event ← queue.peek()
            if t ≥ clock+Δ then
                break
            end if
            match event do
                case Inf(_ → v)
                    OnInfection(t, v, P, queue, border)
                case Rec(v)
                    OnRecovery(t, v, P, queue, border)
            end match
        end while
        clock ← clock + Δ
        for Q in border.project⟨OP⟩() do
            FinishSend(Q)
        end for
    end while
end procedure
```

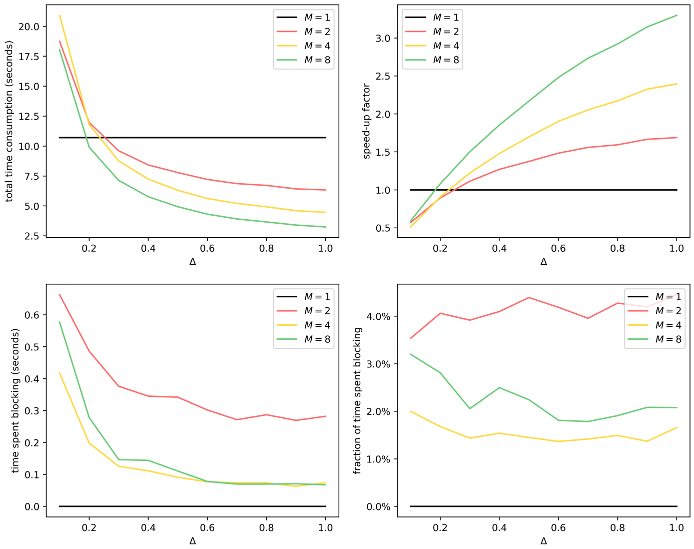

Fig 14. METIS Partitioning of er100k: Average time consumption with different M and Δ. For the $M = 2$ case, the fraction of time spent blocking goes up as Δ goes up, a phenomenon we also observe in fig. 10.

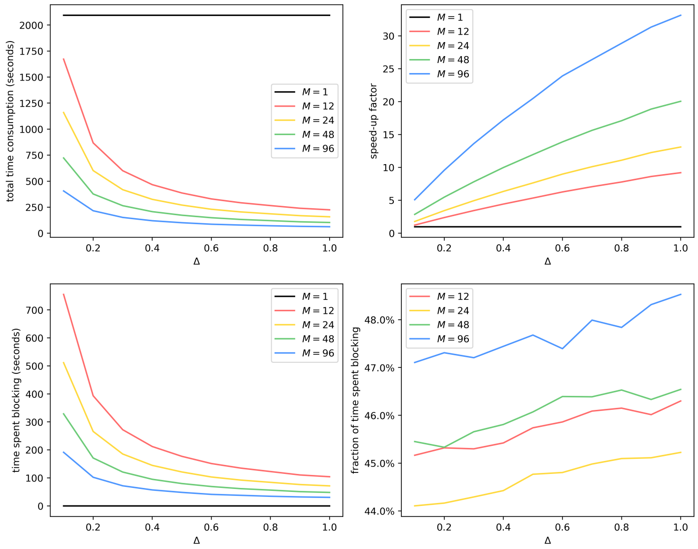

Fig 15. Uniform Partitioning of lj4m: Average time consumption with different M and Δ. The change in the fraction of time spent blocking as Δ goes from 0.1 to 1.0 is around 1% as opposed to the $> 10\%$ changes we see in fig. 10.

all $i \in \{1, 2, \ldots, M\}$ and $r \in \{1, 2, \ldots, R\}$, $R = 10$. The process computation time on P_i, U_i, is computed as

$$U_i = \frac{1}{R} \sum_{r=1}^{R} U_i^r.$$

For each (M, Δ) pair, we compute the coefficient of variation $cv = \sigma/\mu$, where

$$\mu = \frac{1}{M} \sum_{i=1}^{M} U_i, \quad \sigma = \sqrt{\frac{1}{M} \sum_{i=1}^{M} (U_i - \mu)^2}.$$

The result is shown in fig. 16.

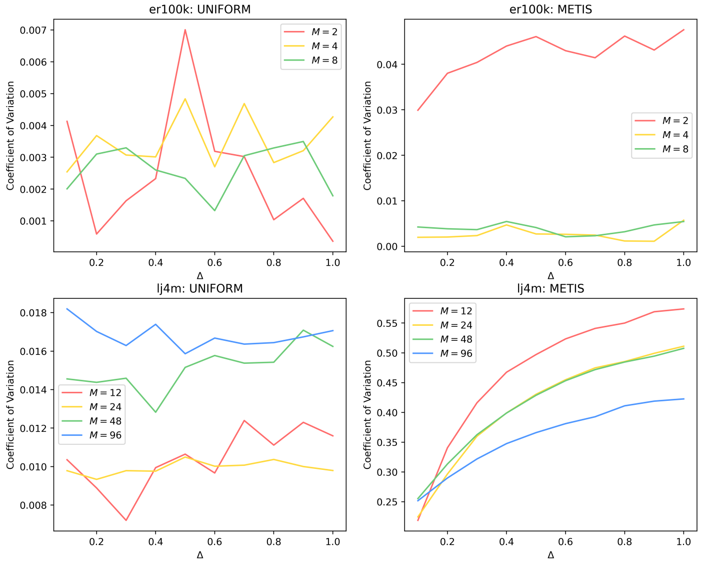

Fig 16. Comparison between er100k and lj4m: Coefficients of variation with different M and Δ. We readily see that when METIS partitioning is used, coefficients of variation go up by an order of magnitude compared to uniform partitioning, and coefficients of variation tend to go up as Δ goes up. In general, the coefficient of variation of process workload is a strong predictor of the fraction of time spent blocking.

We conclude that using a min-cut graph partitioning algorithm can impair load balancing, which causes more time wasted blocking.
