# Supplementary material for: Scalable parallel and distributed simulation of an epidemic on a graph
Source: PLoS One. 2023 Sep 29;18(9):e0291871. doi: 10.1371/journal.pone.0291871 (PMC10540973; doi:10.1371/journal.pone.0291871)
Supplement: Attachment — Submitted filename: Response_to_Reviewers.pdf [file pone.0291871.s002.pdf]

Response to Reviewers

Guohao Dou

Aug. 2023

1 Response to Reviewer #1

1.1 "There is a lack of clarity in Figures 1 and 2 pipeline-like scheme would provide a better understanding of the model's mechanisms"

I agree. In the revised manuscript, figure 2 has been greatly expanded to reflect the mechanism of the method, reproduced below for easy reference.

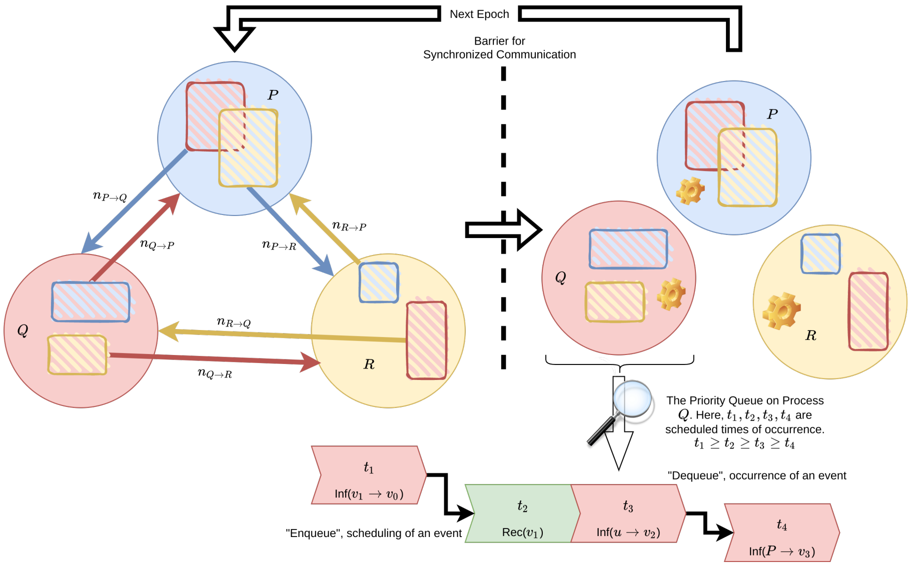

1.2 "The novelty of the proposed technique can be presented better for understanding the readers"

Indeed. We expand both the abstract and the introduction to stress the novelty. Abstract reproduced below for easy reference.

We propose an algorithm to simulate Markovian SIS epidemics with homogeneous rates and pairwise interactions on a fixed undirected graph, assuming a distributed memory model of parallel programming and limited bandwidth. This setup can represent a broad class of simulation tasks with compartmental models. Existing solutions for such tasks are sequential by nature. We provide an innovative solution that makes trade-offs between statistical faithfulness and parallelism possible. We offer an implementation of the algorithm in the form of pseudocode in the Appendix. Also, we analyze its algorithmic complexity and its induced dynamical system. Finally, we design experiments to show its scalability and faithfulness. In our experiments, we discover that graph structures that admit good partitioning schemes, such as the ones with clear community structures, together with the correct application of a graph partitioning method, can lead to better scalability and faithfulness. We believe this algorithm offers a way of scaling out, allowing researchers to run simulation tasks at a scale that was not accessible before. Furthermore, we believe this algorithm lays a solid foundation for extensions to more advanced epidemic simulations and graph dynamics in other fields.

1.3 "Authors should summarize their experiment results in the abstract"

Great catch. Summary of experiment results added to the abstract. See 1.2 for the reproduced abstract.

1.4 "Can the proposed method be combined with graph neural networks [1]. Please include some discussion"

An additional "Related Work" section has been appended to the Introduction section, discussing the possibility of combining the simulation algorithm with graph neural networks, among others.

2 Response to Reviewer #2

2.1 "One concern is that the real dataset used for evaluation comes from a social network, not global epidemics, raising doubts about its suitability and generalizability for epidemic simulation"

Epidemic data is notoriously difficult to come by due to privacy reasons. Also, the main goal of the paper is to articulate the algorithm, instead of establishing empirical results.

2.2 "the paper could benefit from providing more comprehensive background knowledge on susceptible-infected-susceptible models"

Great suggestion. We have edited the introduction to give an intro on SIS model, repreduced below:

> In an SIS model, each individual, represented as a vertex in the graph, can either be in an *infected* (INF) state or a *susceptible* (SUS) state. Susceptible vertices neighboring infected vertices may transition into the infected state through an *infection* event. Infected vertices may transition into the susceptible state through a *recovery* event.

We have also included some key articles to refer to on this classic topic in the "Related Work" subsection.

2.3 "The paper provides direct comparisons with the TDS algorithm, but it does not include direct comparisons with the EDS method, which forms the basis of the proposed algorithm"

In fact, when the number of partitions is one ($M = 1$ for our notation), our algorithm is equivalent to classic sequential EDS. As a result, whenever we have $M = 1$ in our experiments, we are making a comparison with EDS.

It is a good point to point out the equivalence between $M = 1$ and EDS though. We thus add the following sentence in the introduction section:

> In fact, when the number of partitions is one ($M = 1$ as we will see later), our algorithm is reduced to classic sequential EDS, which we use as a baseline in our experiments.

2.4 "Consider adding a "Related Work" section to provide context and comparisons with prior research"

The "Related Work" section has been appended to the Introduction section.

2.5 "Consider introducing initial vertex states INF and SUS with a brief explanation"

Great catch. We have fixed it at the beginning of the Problem Setup section, reproduced below:

> a fixed undirected graph $G = (V, E)$ with initial vertex states (INF or SUS); INF for infected and SUS for susceptible

The two roles are explained in the Introduction section too.

2.6 “Consider renaming section 2 to more detailed titles such as “Problem Setup,” “Method,” “Proofs,” “Experiments,” “Discussion,” and “Results.””

I thought it is required by the template to have an umbrella section called “Results”. Apparently this is not a hard requirement. It has been removed henceforth.

2.7 “Consider adding a few words on the motivation in the abstract to engage readers”

The abstract has been expanded accordingly.

2.8 “Suggest further polishing the paper and addressing any grammar errors to enhance the readability”

Various small fixes have been done.
